# The role of cuproptosis in gastric cancer

**DOI:** 10.3389/fimmu.2024.1435651

**Published:** 2024-10-30

**Authors:** Yixian Li, Wenhao Sun, Shaolin Yuan, Xinxin Liu, Ziqi Zhang, Renjun Gu, Pengfei Li, Xin Gu

**Affiliations:** ^1^ Nanjing University of Chinese Medicine, the First Clinical Medical College, Nanjing, Jiangsu, China; ^2^ Department of General Surgery, Affiliated Hospital of Nanjing University of Chinese Medicine Jiangsu Province, Nanjing, Jiangsu, China; ^3^ Department of Endocrinology, Affiliated Hospital of Nanjing University of Chinese Medicine, Nanjing, Jiangsu, China; ^4^ School of Chinese Medicine, Nanjing University of Chinese Medicine, Nanjing, China; ^5^ Department of Clinical Laboratory, Affiliated Hospital of Nanjing University of Chinese Medicine, Jiangsu Province Hospital of Chinese Medicine, Nanjing, Jiangsu, China

**Keywords:** cuproptosis, gastric cancer, mechanism, immune, therapies

## Abstract

As a biologically essential transition metal, copper is widely involved in various enzymatic reactions and crucial biological processes in the body. It plays an increasingly important role in maintaining normal cellular metabolism and supporting the growth and development of the human body. As a trace element, copper maintains the dynamic balance of its concentration in body fluids through active homeostatic mechanisms. Both excess and deficiency of copper ions can impair cell function, ultimately leading to cell damage and death. Cuproptosis is a novel form of cell death where copper ions cause cell death by directly binding to the lipoylated components of the citric acid cycle (CAC) in mitochondrial respiration and interfering with the levels of iron-sulfur cluster (Fe-S cluster) proteins, ultimately causing protein toxic stress. Its primary characteristics are Cu2+ concentration dependence and high expression in mitochondrial respiratory cells. Recent research has revealed that, compared to other forms of programmed cell death such as apoptosis, necrosis, and autophagy, cuproptosis has unique morphological and biochemical features. Cuproptosis is associated with the occurrence and development of various diseases, including cancer, neurodegenerative diseases, and cardiovascular diseases. This article focuses on a review of the relevance of cuproptosis in gastric cancer (GC).

## 1 Introduction

Gastric cancer (GC) is a primary epithelial malignant tumor originating in the stomach. It has various pathogenic factors, and its morbidity and mortality rank among the top five malignant tumors worldwide (1). Due to the stomach being a hollow organ, it provides more space for tumor growth compared to other solid organs. Additionally, the early symptoms of GC are relatively insidious (2). As a result, GC is often diagnosed at an advanced stage (3), imposing significant economic and health burdens on people.

Cuproptosis is a recently discovered form of programmed cell death triggered by copper overload *in vivo*. The basic principle involves copper ions interfering with the levels of Fe-S cluster proteins by directly binding to the lipoylated components of the citric acid cycle (CAC) in mitochondrial respiration, causing proteotoxic stress, and ultimately leading to cell death (4). This article reviews the relevance of cuproptosis in GC and provides insights and suggestions for the prevention and treatment of GC from the perspective of the cuproptosis mechanism.

Gastric cancer (GC) is a primary epithelial malignant tumor originating in the stomach. It has multiple pathogenic factors, and its morbidity and mortality rank among the top five malignant tumors worldwide (1). Due to the stomach being a hollow organ, it offers more space for tumor growth compared to other solid organs. Additionally, the early symptoms of GC are relatively insidious (2), often resulting in late-stage diagnosis (3). This delay in diagnosis imposes significant economic and health burdens on individuals. Cuproptosis is a recently discovered form of programmed cell death triggered by copper overload *in vivo*. The underlying mechanism involves copper ions interfering with the levels of Fe-S cluster proteins by directly binding to the lipoylated components of the citric acid cycle (CAC) in mitochondrial respiration. This interaction causes proteotoxic stress, ultimately leading to cell death (4). This article reviews the relevance of cuproptosis in GC and provides insights and suggestions for the prevention and treatment of GC from the perspective of the cuproptosis mechanism.

## 2 Association between GC and programmed forms of cell death

With the significant improvement in the living environment and the reduction of bad living habits (1), the incidence and mortality rates of GC in China are decreasing year by year (2). However, since most GC patients are incurable, their survival rate is low (3), which poses a serious threat to people's health as well as a heavy medical burden to society (4, 5). As a common malignant tumor of the digestive system (6), the development of GC is intimately related to disorders of programmed cell death, which is proved by numerous studies (7, 8).

Programmed cell death (9) is generally classified as apoptosis (10), pyrozosis (11), necroptosis (12), autophagy (13), etc. According to relevant studies, among others, apoptosis is considered to have a definite correlation with cancer (14, 15). Programmed cell death is an active and inherently programmed phenomenon (16), it is influenced by the physiological and pathological environment of the body. It plays a major role not only in the maintenance of cellular homeostasis in organisms but also in the normal development of the body (17). Since programmed death is an orderly arranged, differentiated cell growth-death process, it can occur under both physiological and pathological conditions (18). During tissue differentiation, atrophy, or degeneration, it strictly follows an established procedure to inhibit cell proliferation, precisely eliminating cells that are no longer needed by the organism and becoming one of the organism's defenses against disease. Moreover, it is important to note that it can also maintain the normal state of various physiological activities of the body by actively inducing cell death (19–22). Its extraordinary property brings new hope to the current dilemma of preventing and combating cancer.

With the significant improvement in living conditions and the reduction of harmful lifestyle habits (5), the incidence and mortality rates of gastric cancer (GC) in China are decreasing year by year (6). However, since most GC cases are diagnosed at an incurable stage, the survival rate remains low (7), posing a serious threat to public health and imposing a heavy medical burden on society (8, 9). As a common malignant tumor of the digestive system (10), the development of GC is closely linked to disorders of programmed cell death, as evidenced by numerous studies (11, 12). Programmed cell death (13) is generally classified into apoptosis (14), pyroptosis (15), necroptosis (16), autophagy (17), and other types. Among these, apoptosis has a well-established correlation with cancer, according to relevant studies (18, 19). Programmed cell death is an active and inherently programmed phenomenon (20) influenced by the physiological and pathological environment of the body. It plays a crucial role in maintaining cellular homeostasis and the normal development of the body (21). As an orderly, regulated process, programmed cell death can occur under both physiological and pathological conditions (22). During tissue differentiation, atrophy, or degeneration, it strictly follows an established procedure to inhibit cell proliferation, precisely eliminating cells that are no longer needed by the organism, thus serving as one of the body's defenses against disease. Furthermore, it actively induces cell death to maintain the normal state of various physiological activities in the body (23–26). This extraordinary property brings new hope to the current challenges in preventing and combating cancer.

The infinite growth of gastric malignant tumor cells is the consequence of the inhibition of programmed tumor cell death (23). Once the cell death process is disrupted or inhibited, tumor cells will grow indefinitely, ultimately leading to cancer (24). Cuproptosis is a novel form of cell death discovered and named in March 2022 by Tsvetkov et al. That study found that copper ions can impair the function of mitochondrial enzymes in the CAC via the FDX1 gene when copper ion levels in the body exceed normal values in humans. The mitochondrial membrane is then subjected to oxidative damage and binds directly to the lipid-acylated component of the CAC. Thereafter, they form long chains or clustered aggregates and interfere with iron-sulfur clusters, causing iron-sulfur proteins to be down-regulated. This contributes to proteotoxic stress, which ultimately causes cell death (25).

Cuproptosis is a new format of cell death distinct from oxidative stress-related forms of cell death (such as apoptosis, iron death, and necrotic apoptosis). Copper ions, as key metal ions for cell signaling, can be extensively involved in cancer development by promoting cellular value-addition, angiogenesis, and metastasis (26). There is now substantial clinical evidence of the efficacy of using copper homeostasis in the treatment of cancer (27).

Therefore, this article will review the research progress on the role of cuproptosis in GC occurrence and development, and perspective in this regard, to provide new perspectives for exploring the pathogenesis and potential therapeutic targets of GC.

## 3 Gastric cancer

The uncontrolled growth of gastric malignant tumor cells results from the inhibition of programmed tumor cell death (27). When the cell death process is disrupted or inhibited, tumor cells can grow indefinitely, ultimately leading to cancer (28). Cuproptosis is a novel form of cell death discovered and named by Tsvetkov et al. in March 2022. Their study found that copper ions can impair the function of mitochondrial enzymes in the citric acid cycle (CAC) via the FDX1 gene when copper ion levels in the body exceed normal values. The mitochondrial membrane then undergoes oxidative damage and binds directly to the lipoylated components of the CAC. This interaction forms long chains or clustered aggregates and interferes with iron-sulfur clusters, leading to the downregulation of iron-sulfur proteins. This causes proteotoxic stress, ultimately resulting in cell death (29). Cuproptosis is distinct from other forms of cell death related to oxidative stress, such as apoptosis, ferroptosis, and necroptosis. Copper ions, as key metal ions for cell signaling, can be extensively involved in cancer development by promoting cellular proliferation, angiogenesis, and metastasis (30). There is now substantial clinical evidence supporting the efficacy of targeting copper homeostasis in cancer treatment (31).

GC is a disease of high molecular and phenotype heterogeneity, which is driven by multiple genetic and epigenetic aberrations (28). The most common histological type is gastric adenocarcinoma(GA) originating from the epithelium of the gastric mucosa. It accounts for more than 95% of GC cases (29). That is also the point of this article. The Lauren classification and the WHO classification are routinely used in clinical practice, to distinguish different histological and molecular biological characteristics in GC patients (30). Based on the diversity of glandular structures, Lauren's classification divides GC into intestinal gastric cancer, diffuse gastric cancer, and mixed GC (31). IGC, DGC, and MGC exhibit distinct clinical features (32) and genetics of specificity (33, 34), individually. Notably, the WHO classification, which is based on cellular morphology and histological structure, is a refinement and addition to Lauren's classification. This classification system involves papillary, tubular, mucinous, and MGC (35).

IGC commonly forms one or several solid tumors (31, 36), mostly due to Helicobacter pylori infection (37), and is seen as damage to the gastric glands. In contrast, the DGC consists of many disjointed microscopic tumors (31, 38). DGC is typically characterized by ill-defined or absent glandular structures and reduced intercellular adhesion, compared to IGC (39). As a consequence, they are more aggressive which leads to a worse prognosis. Furthermore, IGC and DGC respectively consist of diverse cellular lineages. In IGC, tumor cells normally evolve from gastric intestinal metaplasia (GIM), with a progressive increase in tumor cell heterogeneity as the malignant stage progresses. While in the cell lineage of DGC, cancer cells are derived from tumor stem cells (TSCs) (40). Studies have shown that peripheral stem cells can transiently outperform average growth regulation mechanisms, tissue regeneration, and growth in complex situations, thereby increasing the risk of cellular mutation. And it is known that TSCs have similar traits to peripheral stem cells. TSCs can interact with cancer-associated fibroblasts (CAFs) within the tumor and evolve into distinct cell populations, contributing to the onset of DGC heterogeneity and eventual progression to GC (41). Based on proteomics analysis, DNA damage was identified to be dramatically upregulated in IGC. As a crucial kinase for DNA mismatch repair, ATM/ATR can regulate cell proliferation in IGC, through activation of the SWI/SNF complex, whereas immunity and up-regulation of extracellular matrix proteins (ECMs) are seen in DGC (42).

As opposed to IGC, which tends to metastasize to solid organs (liver, lungs), DGC frequently metastasizes via lymph nodes and ultimately spreads to the intra-peritoneum (43). DGC commonly has a stronger affinity for intestinal neurons than IGC and has a stronger perineural infiltration rate (44). In review, we considered that IGC and DGC share unique histological and pathological features, and the heterogeneity between the roles of the two may be associated with their independent cellular lineage characteristics. Since they are distinguished by different cellular lineages, we can provide specific targeted therapy for GC patients. Nevertheless, we still have a large rising range for the study of IGC and DGC in the mechanism of tumor cell evolution.

Since the stomach is a hollow organ and the early symptoms of GC are insidious (45). For this reason, the majority of GC have usually progressed to advanced stages and have a high mortality rate, by the time they are diagnosed, causing it to be the fourth most common cause of cancer-related deaths after liver cancer. In 2020, the worldwide number of GC patients reached 1.09 million, with 770,000 deaths, contributing to 5.6% and 7.7% of the global cancer incidence and mortality rates, respectively (46). While the incidence and mortality rates of GC are currently declining, in most countries, owing to economic growth and the widespread concept of disease prevention, the deaths from GC will only increase in the future as the population aging progresses (47).

Family history, Helicobacter pylori infection, GIM (48), genetic factors, and environmental and dietary factors (49). are the most prevalent etiological factors. Chronic atrophic gastritis (AG) and GIM are widely recognized as common precancerous lesions. Furthermore, H. pylori invasion has been proved to be an essential element to cause AG and GIM. The relationship between H. pylori and GC is influenced by both the severity and rate of progression of AG. A chronic inflammatory process in the gastric mucosa, combined with damage to the gastric glands (50), and a decrease in gastric secretory function characterize AG.

H. pylori not only colonizes and survives in the human digestible tract, but also enhances its capacity to cause disease by infection. Since H. pylori is a Gram-negative bacterium with a specific flagellar structure and secretion of urease that can neutralize gastric acid and other properties (51).

Additionally, on account of genome plasticity, the genome sequence of H. pylori can change by more than 20% to modify to a specific host environment during colonization (52). The main virulence factors of H. pylori are Vacuolating cytotoxin A (Vac-A), cytotoxin-associated gene A (Cag-A), cytotoxin-associated gene pathogenicity island (Cag-PAI), and bacterial flagella. H. pylori Vac-A has a variety of cellular actions, including the utilization of cellular vacuoles to increase the lifespan of infection, as well as mitochondrial stress, and interference with apoptosis (53), Vac-A also regulates host cell metabolism, stimulating all three of these pathways, including NFE2L2 / HO-1 /Nrf2-HMOX1, through the dependent inhibition of MTORC1 (54). Thereby further inducing autophagy by perturbing mitochondria and depleting cellular amino acids in human GC cells. This ultimately provides the nutrients and intracellular ecological niche necessary for H. pylori to colonize and replicate on its own (55). If this autophagy is dysfunctional, it can damage tight junction proteins and disrupt the integrity of the gastric epithelial barrier. This effect can contribute to the development of primary gastrointestinal diseases by severely affecting intestinal homeostasis and the host's inflammatory response (56). Excessive ROS produced by autophagy in H. pylori has also been identified as a potential factor in its induction of GC incidence via autophagy. Some related research has found that the T4SS, encoded by the Cag-PAI, also injects Cag-A into gastric epithelial cells (57, 58). Such changes cause genomic instability of gastric epithelial cells, promotion of inflammation and malignant transformation of epithelial cells, as well as sustained proliferation of tumor cells (59).

These virulence factors in H. pylori also interfere with the host's intercellular signaling cascade, causing tumor cells to escape growth-inhibiting factors and resist cell death. Not only that, it can adhere to gastric epithelial cells (60)by binding to receptors via a series of outer membrane proteins, Including adhesion lipoproteins A and B (AlpA / B), blood group antigen binding adhesion (BabA), etc. Typical symptoms of acute-chronic inflammation can arise from persistent H. pylori infection and damage to the gastric acid-producing glands. Through the above mechanisms, AG is eventually caused. Continued AG will destroy and kill the acid-producing wall cells, resulting in too little or a lack of stomach acid production. This weakened acidic environment accelerates the colonization of a pro-inflammatory gastric microbiota. This harmful microbiota produces additional genetically toxic pro-inflammatory metabolites and carcinogens. The above ultimately led directly to the transformation of the malignant epithelial cells of the stomach (60–62), leading to the formation of GC (49) , H. pylori infection is still considered a major causative factor in GC, although the virulence of its carcinogenic is influenced by multiple microbiological, environmental, and host factors (63). Whereas widely dispersed AG is known to be associated with the induction of a state of gastric acid deficiency or hypogastric acidity, which is one of the significant risks for the development of GC (64, 65).

In addition, inactivation of tumor-inhibiting genes caused by DNA hypermethylation plays an influential position in the occurrence and progression of GC (66–69). It has been extensively demonstrated that factors associated with GC development, such as protocadherin 10 (PCDH10) (70), and junctional adhesion molecule 3 (JAM3) (71), lead to down-regulation of tumor suppressor genes (TSGs) through promoter hypermethylation in GC, are not infrequent in a wide range of GC types. However, the specific mechanisms involved need to be further elucidated. Regarding the eradication of H. pylori, partial studies have found that it reverses the methylation process of the E-calmodulin gene in patients with chronic gastritis. It has also been demonstrated in specific genes that the methylation levels of other TSGs are reduced after this treatment (72).

GIM refers to the replacement of the gastric epithelial mucosa by intestinal epithelial mucosa of the Penn cell, absorptive enterocyte, and cuprocyte types (73–75). According to the histological classification, “complete GIM” is known as “type I” or “intestinal type”, and “incomplete GIM” is known as “colonic type” and includes “type II” and “type III”. In comparison, the latter has a higher risk of cancer (76–78). According to the classic Correa cascade theory (79). the development of GC involves multiple stages, from mild to severe, healthy gastric mucosa, superficial gastritis, AG, GIM, heterogeneous hyperplasia, and finally malignant transformation. The first step in this cascade is the emergence of chronic gastric mucosal inflammation mediated by polymorphonuclear cells and mononuclear cells. This stage causes multifocal glandular atrophy in the stomach, loss of cell mass in the gastric wall, and loss of acid secretion in the stomach. As it continues to atrophy, the gastric epithelium is progressively replaced by an intestinal-type epithelium, mainly characterized by the inclusion of Pannus cells, absorptive enterocytes, and cup cells. GIM is considered to be an irreversible turning point in GC, and progression from GIM to low-grade atypical hyperplasia, high-grade atypical hyperplasia, and intestinal-type GA is extremely probable (80). With atrophy of the intestinal-type mucosa and progression of GIM, heterogeneous hyperplasia (intraepithelial neoplasia) can occur, eventually progressing to gastric malignancy (81). As a precancerous lesion, GIM plays an essential role in the development of GC, and it signals a high risk of GC.

It is widely accepted that gastric mucosal epithelial cells are subject to multifactorial influences that produce genetic mutations that activate proto-oncogenes or silence oncogenes. This mutation will destroy the balance between cell proliferation and programmed cell death, ultimately resulting in GC. Although this became the existing consensus, the specific molecular mechanisms of gastric carcinogens have not been fully revealed (82, 83).

As early symptoms of GC are not noticeable, once the diagnosis is confirmed, it is often in the middle to late stages and the prognosis is dire (84).

Therefore, it becomes exceedingly important to seek effective early diagnosis of GC and corresponding treatment modalities.

Copper is an indispensable trace metal element involved in various physiological processes in the human body. It has been shown in numerous studies that serum and tumor tissue concentrations of copper ions are dramatically increased in cancer patients compared to healthy individuals (85, 86).

In 2022, Tsvetkov et al. identified a new form of programmed cell death, copper concentration-dependent cell death, also known as cuproptosis. They have shown that Cu^2+^ binds directly to the lipoylated components of the TCA and that the aggregation of these copper-bound lipoylated mitochondrial proteins. The consequent loss of Fe-S-cluster proteins triggers proteotoxic stress, ultimately leading to cell death (87).

## 4 Cuproptosis

Cuproptosis is a recently discovered form of programmed cell death triggered by copper overload *in vivo* (88). In 2022, Tsvetkov et al. found that when blocking known modes of cell death (e.g. apoptosis, autophagy, iron death, etc.), molecules or ion carriers bound to copper ions were still able to trigger cell death in unique yet similar ways. Based on metabolomics, they identified more CAC-related metabolites in copper-sensitive cells, further substantiating that Cu^2+^ plays an important role in the CAC. The above results indicate that the cell-killing effect of copper overload is likely to be related to the process of mitochondrial respiration. And that components of the CAC are essential targets of cuproptosis (25).

Further studies by Tsvetkov et al. illustrated that cells utilizing mitochondrial energy production were nearly 1,000 times more sensitive to copper carriers than cells utilizing glucose glycolysis production. Using a CRISPR knockout screen, the team identified several key genes that promote copper death, including the FDX1 gene, which encodes the target protein of the Elesclomol molecule, and six genes involved in mitochondrial metabolism and protein fatty acylation modification. These six genes are DLAT, PDHA1, PDHB and DBT, GCSH, and DLST. A study found that reductase, encoded by the FDX1 gene, converts Cu^2+^ to the more toxic Cu^+^ and may provide a direct Elesclomol target (89).

**Figure 1:**
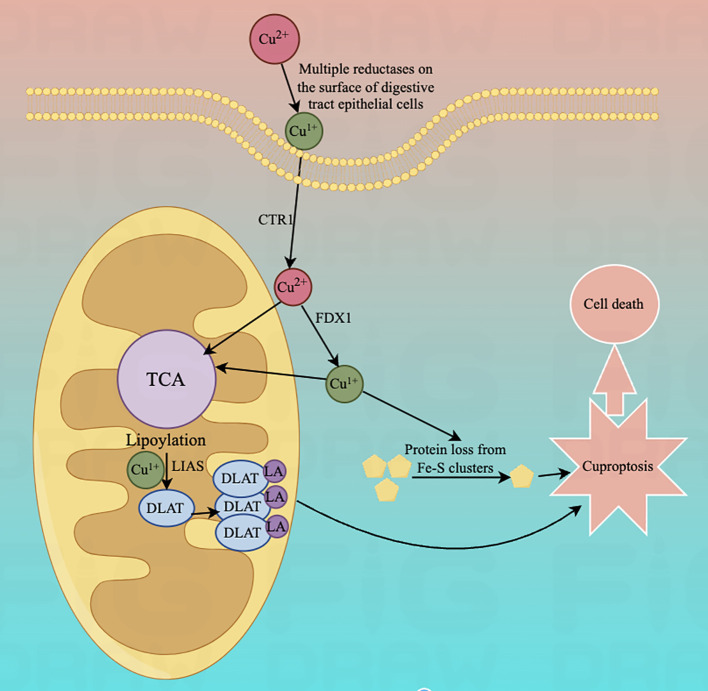
Diagram of the mechanism associated with cuproptosis.The copper ion binds to thioctylated proteins in the tricarboxylic acid cycle (TCA) and promotes aberrant oligomerization of thioctylated proteins. At the same time, copper ions can also reduce the level of Fe-S cluster proteins, which together induce a proteotoxic stress response that ultimately leads to cell death.

Lastly, for exploring the link between copper toxicity and protein lipid acylation, to identify specific metabolic pathways that mediate copper toxicity. Todd Golub's team postulated that copper might bind directly to isolated proteins. In their experiments, they found that cuproptosis is strongly aligned with mitochondrial metabolism-mediated protein-lipid acylation processes. This also values FDX1 and Protein Lipidation as crucial regulators of cuproptosis, with FDX1 being an upstairs regulator of the latter. This experiment suggests that the protein's thioctyl portion is necessary for copper binding. Copper ions penetrating mitochondria via copper carriers directly bind to these lipoylated modified proteins and induce oligomerization of DLAT, resulting in their formation of long chains and clustering. Alternatively, copper interferes with Fe-S clusters and induces loss of Fe-S cluster proteins, leading to the elevation of HSP70 to activate acute proteotoxic stress that leads to cell death. All results indicate that excess copper promotes aggregation of lipid-acylated proteins and instability of Fe-S cluster proteins, ultimately leading to cytotoxic stress leading to cell death (**Figure 1**).

Overall, this study presented cuproptosis, a novel copper concentration-dependent cell death modality, and further revealed its specific mechanism.

Currently, research on cuproptosis is progressing in a number of non-cancer diseases, in addition to advances in the field of cancer. Examples include cardiovascular disease, inflammatory responses, and neurodegenerative diseases.

A meta-analysis has shown that serum copper levels are elevated in patients with type 2 diabetes compared with healthy controls (90). Because impaired pancreatic β-cell function is closely related to cell death in patients with type 2 diabetes. Therefore, the concept of cuproptosis also provides new ideas for the study of copper metabolism and potential therapeutic mechanisms in diabetes (91). In addition, it has been confirmed that protein toxic stress associated with cuproptosis is a potential factor in the pathogenesis of many cardiovascular diseases (92). In addition, due to the relevance of cuproptosis to cellular mitochondrial function and oxidative stress pathways, studies based on the concept of cuproptosis to reduce cell damage related to cardiovascular disease have attracted increasing attention. Moreover, recent progress has been made in the correlation between cuproptosis and inflammatory response. Studies have shown that the body's inflammatory response is closely linked to cellular mitochondrial dysfunction (93). Researchers have found that significant mitochondrial fragmentation is seen in septic endothelial cells, which may be associated with reduced mitochondrial membrane potential and increased reactive oxygen species production (94). ZhangJun's team constructed a predictive model and found a significant correlation between the expression of the key cuproptosis genes PDHB, PDHA1, and LIAS (95), which are mainly found in cellular mitochondria and function as the catalytic subunit of pyruvate dehydrogenase (PDH) (96), and the prognosis of sepsis patients. Since PDH activity is often inhibited in patients with sepsis (97), modification of PDHB and PDHA1 seems to significantly influence the development of sepsis. As a key gene encoding the mitochondrial lipoic acid pathway (98), LIAS is also important for the prognosis of sepsis patients. Wilson Disease is an inherited disease of the liver characterized by an overload of copper in liver tissue. Since the maintenance of copper content in the liver in equilibrium is mediated by the ATP7B protein encoded by the ATP7B gene. Therefore, once the ATP7B gene is expressed abnormally, it leads to impaired synthesis of ceruloplasmin (the major copper-containing protein in the blood) in the liver as well as impaired biliary excretion process of copper in the liver, which ultimately leads to overaccumulation of copper in the liver. Since ATP7B is also a key gene leading to the occurrence of cuproptosis in cells, cuproptosis has been recognized as the pathogenesis and new therapeutic breakthroughs of Wilson Disease in new research reports (99). In addition, it has been shown that one of the major causes of brain neurological damage due to dysregulation of copper homeostasis is the induction of the Haber-Weiss and Fenton reactions by copper in its oxidation state and reduction state circulatory transitions, resulting in the production of excessive ROS products that cause oxidative damage to proteins, lipids, and DNA. This induces oxidative damage to proteins, lipids, and DNA, ultimately leading to neurotoxicity and cellular dysfunction (100). Because the occurrence of cuproptosis is affected by proteins related to copper ion transport, storage and uptake pathways, such as SLC31A1, ATP7A, ATP7B, etc, recent studies related to cuproptosis and neurodegenerative diseases are attracting the attention of researchers (101).

## 5 The relationship between GC and cuproptosis

### 5.1 Cu^2+^ and GC development mechanism

As an indispensable enzyme coordinator in the human body, a variety of studies have documented abnormal copper concentrations in the serum of patients with many malignant tumors, such as gastric, breast, brain, prostate (102), colon, lung, and liver cancers, to varying degrees (103–110). Copper has a strong coordination capacity. Excessive copper will form complexes in the body with amino acids, proteins, or other substances that interact with enzymes, nucleic acids, DNA, and other macromolecules, leading to malignant cell differentiation. The significant increase of Cu level will promote the catalytic effect of copper-mediated in mitochondria, resulting in the generation of reactive oxygen species (ROS), and excessive ROS will lead to the oxidation of the amino acid side chain of the protein or the break of the peptide chain, which will change the properties of the protein and lead to the loss of various enzyme activities. ROS may oxidize the bases of DNA or degrade DNA; In addition, ROS may also act on unsaturated fatty acids in cell membranes, causing lipid peroxidation and disrupting the normal function of membranes. Substantial evidence exists that unbalanced copper homeostasis can influence tumor growth (111). This promotes the growth and spread of tumor cells in the body.

Although it has been extensively demonstrated and accepted that the occurrence and development of GC are highly correlated with abnormal programmed cell death in the gastric mucosa (112). There is uncertainty about the specific mechanisms underlying the abnormal value-added of GC cells.

There is evidence that cancer cells generally have stronger copper requirements than healthy resting cells. Compared to other tumor cells, GC cells prefer to employ glycolysis to produce intermediate metabolites and energy to increase resistance to cuproptosis (113). Also, as a vital element in cell signaling, copper ions are involved in the activation of cell proliferation-related signaling pathways. It is involved in cancer occurrence and progression by promoting cell proliferation, angiogenesis, and metastasis (114) Analyzing the TCGA database, we discovered a remarkable functional enrichment of lncRNA genes associated with cuproptosis in the inflammatory response, immune response, and transmembrane cell signaling in GC. The genes CD209 and HAVCR2 are cuproptosis-associated regulatory immune checkpoints. When they are overexpressed in GC tissue, this means that GC patients have a lower survival rate (115). Associated studies have uncovered that cuproptosis-related genes including FDX1, ENTPD3, PDZD4, CNN1, GTPBP4, FPGS, UTP25, CENPW, and FAM111A. They can accurately predict the progression of GC and have excellent performance in the early diagnosis and treatment of GC. Among these, FDX1 has been shown to induce cuproptosis by an atypical methyltransferase called METTL16 by modifying FDX1 mRNA (116). There is increasing evidence that lncRNA implicated in cuproptosis can be utilized as biomarkers of prognosis in GC patients. There is increasing evidence that lncRNA implicated in cuproptosis can be utilized as biomarkers of prognosis in GC patients (117).

**Figure 2:**
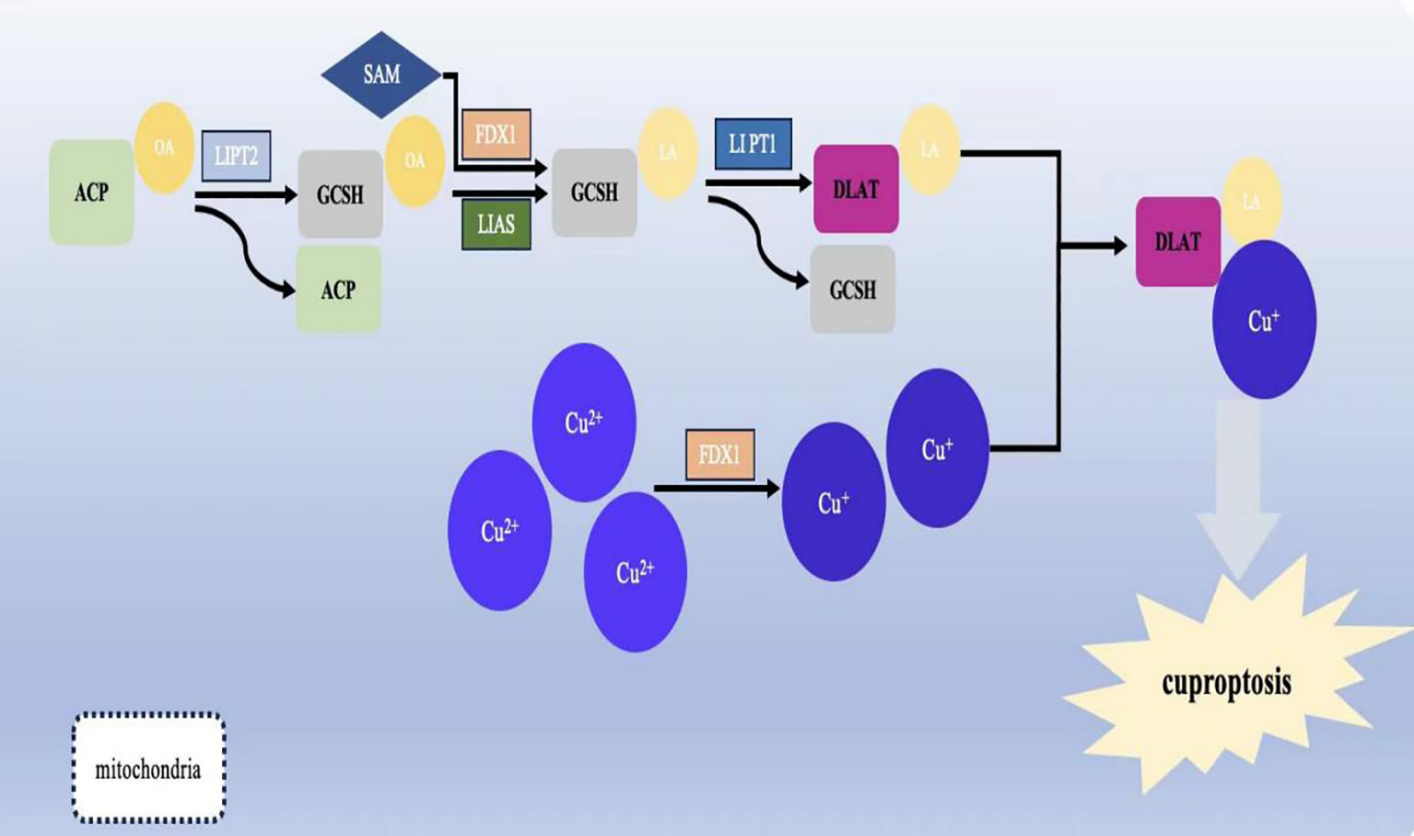
Schematic representation of the mechanism of cuproptosis and its effect on mitochondrial function.Lipoylation is required for the activation of several mitochondrial enzyme complexes, and the biosynthesis of lipoic acid in humans involves several steps: the acyl chain on the acyl-carrier protein (ACP) generates octanoyl ACP through extension, reduction, and dehydration; lipolytransferase 2 (LIPT2) transfers the octanoyl portion from ACP to the Glycine cleavage system H protein (GCSH); LIAS generates ACP by depleting S-adenosyl methionine (SAM); and LIAS generates ACP by depleting SAM. Lipolytransferase 2 (LIPT2) transfers the octanoyl portion from ACP to Glycine cleavage system H protein (GCSH); LIAS completes the formation of lipoic acid on GCSH by depleting SAM and inserting sulphur atoms into carbons 6 and 8 of the octanoyl group; finally, the formation of lipoic acid is completed by the depletion of SAM; the formation of lipoic acid is completed by the depletion of SAM. Finally, LIPT1 transfers the thiooctanoyl portion from GCSH to target proteins (DLAT, DLST, etc.), thus completing the thiooctanoylation of mitochondrial proteins.

In July 2023, Yong fu Shao's team proposed a column-line graph model based on copper death-related genes to predict overall survival and cancer-specific survival in GC patients. The model found that copper death-related genes FDX1, LIASd, and MTF1 could be used as potential prognostic biomarkers for GC patients (118). In 2024, jia-qi jin (119) and others related research also confirmed this conclusion.And XiaoJunYang found that FDX1 was significantly up-regulated in GCtissues. To suppress FDX1 will lead to GC cells malignant phenotype transformation is restrained (120). Approximately 10 genes associated with cuproptosis (121)can be expressed in different types of GC. These include seven positively regulated cuproptosis genes: ferredoxin1 (FDX1), lipoic acid synthase (LAIS), fatty acid transferase 1 (LIPT1), dihydrolipoicenamide dehydrogenase (DLD), dihydrolipoic acid transacetylase (DLAT), pyruvate dehydrogenase E1-alpha subunit (PDHA1), pyruvate dehydrogenase E1-β subunit (PDHB) and three negatively regulated genes: metal-regulated transcription factor 1 (MTF1), glutaminase (GLS), and cell cycle protein-dependent kinase inhibitor 2A (CDKN2A) (see **Table 1** below) (**Figure 2**).

**Table 1:** The markers of Cuproptosis.

Cuproptosis markers	Ways to participate	Reference documentation
FDX1	①Reduction of Cu^2+^ to more toxic Cu+ induces cuproptosis; ②Catalytic lipoylation of the core structural protein of PDH affects the CAC to induce cuproptosis	(122, 123)
DLAT	Catalyzes the conversion of pyruvate to acetyl-CoA and promotes lipid synthesis, which is involved in the CAC and binding to copper ions induces cuproptosis in cells	(89, 124, 125)
PDHB	① Thiooctylated CAC-associated enzymes can promote cell death by directly binding Cu^2+^ ② Binding to copper ions leads to oligomerization of thiooctylation-modified CAC-related enzymes and formation of insoluble aggregates, leading to cytotoxicity and induction of cell death.	(88)
PDHA1	Encodes the α subunit of the PDH complex, which catalyzes the conversion of pyruvate to acetyl-CoA and thus participates in the CAC, binds to copper ions, and induces cuproptosis in cells	(126)
LIPT1	Activates mitochondrial 2-keto acid dehydrogenase participates in lipoylation, binds to copper ions, and engages in cellular cuproptosis	(127)
LIAS	An associated gene as a crucial enzyme of the lipoic acid pathway, regulated by FDX1, is implicated in cellular cuproptosis	(128)
DLD	An associated gene as a critical enzyme of the lipoic acid pathway, regulated by FDX1, affects mitochondrial metabolism, is involved in the CAC, and is implicated in cellular cuproptosis	(129–131)
ATP7A	Engaging in the metabolic pathway of cellular copper Cu^2+^ leads to severe disruption of intracellular content, which in turn leads to cellular cuproptosis	(132)
GLS	GLS is a key enzyme in glutamine metabolism, converting glutamine to glutamate, decomposing it to produce α-ketoglutarate, and entering the CAC to counteract oxidative stress in tumors, thus preventing and treating cuproptosis.	(133)
MTF1	It combines with MRE, activates MTs, and engages in an intracellular overloaded Cu^2+^ transcriptional response to control metal and oxidative stress, which inhibits the onset of cellular cuproptosis.	(134–136)

### 5.2 Cuproptosis markers

Except for the Cuproptosis-related genes(CRGs), which were highly expressed in GC cells (137), Organismal overload of copper ions may also contribute to the occurrence and development of gastric malignancies by regulating the tumor microenvironment (TME) (138).

The initiation and progression of cancer occur almost concurrently with changes in the surrounding stroma. Cancer cells can be functionally shaped to fit the TME for tumor cell growth and development by secreting various cytokines, chemokines, and other factors that lead to reprogramming of the surrounding cells (139). TME is intimately associated with tumor progress and consists of different types of immune, stromal, and immune Cells that are an essential part of it (140, 141).

Studies have shown that the trace element copper, which is important in cellular and humoral immunity, can be used to activate and maintain the immune system by manipulating various immune cells (142, 143). The establishment of crosstalk between cancer cells and proximal immune cells will provide nutritional support to tumor cells and promote cancer development and progression. They will eventually turn into a TME suitable for tumor growth and metastasis (144, 145). Therefore, an in-depth comprehension of the features of copper overload-mediated TME immune cell infiltration will help us better know the potential mechanisms of GC, predict the response to immunotherapy, and develop new safe and efficient targeted drugs (146).

Additionally, angiogenesis is an influential factor in tumor progression. Blood vessels rarely form new branches under physiological conditions in healthy adults. In contrast, in cancerous tissue, without a blood vessel supply, tumors cannot grow beyond 1-2 mm (147). Tumor angiogenesis is a sophisticated process engaging endothelial cell migration and proliferation, as well as vascular tube and new vessel generation. Copper ions are thought to initiate angiogenesis (148). Excess copper stabilized HIF-1α, the rate-limiting component of HIF-1, causing its accumulation in the cytoplasm, which activated HIF-75, regulated vascular endothelial growth factor(VEGF)expression, and promoted tumor angiogenesis (148).

**Figure 3:**
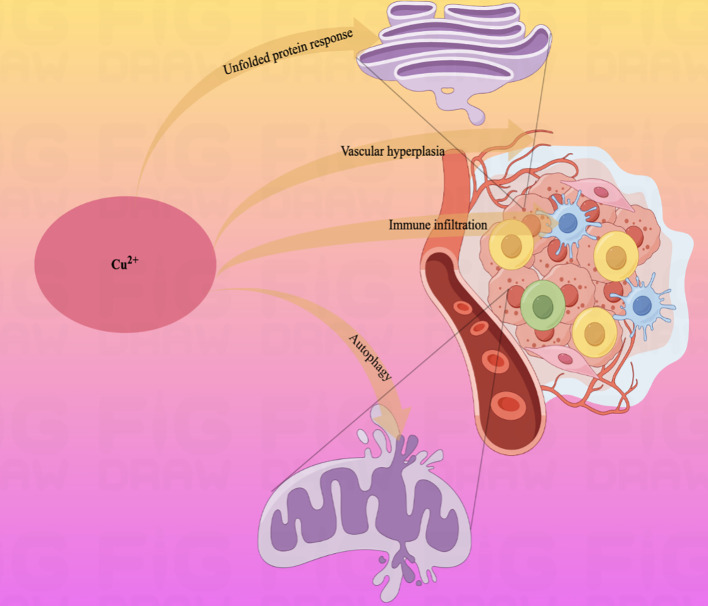
Schematic diagram of the pathways by which copper ions affect some physiological activities of the human body.Copper ions are widely involved in the physiopathological activities of the body through autophagy, vascular proliferation, and immune infiltration.

Moreover, tumor cells commonly have abnormal mitochondrial metabolism due to the loss of active oncogenes and tumor suppressor genes (149). As a metal nutrient, there is a consistently high demand for copper ions during tumor growth and metastasis. Combined with the high expression of Cuproptosis in mitochondrial respiratory cells, it is evident that copper-related diagnostic and therapeutic approaches are appropriate for gastric malignant tumors (**Figure 3**).

### 5.3 Treatment of cuproptosis and GC

GC treatment options are selected with reference to several elements: tumor stage, biomarkers, and the doctor's preferred option (150). For early-stage cancers, clinical emphasis is put more on tumor resection surgery (151) than comprehensive systemic treatments like chemotherapy or radiotherapy. Nevertheless, it is not unusual for patients with GC to opt for integrative therapies in the clinic, as GC is often at an advanced stage at the time of diagnosis.

Studies have shown that copper levels and lactate concentrations in GC tissues are significantly higher than in normal gastric tissues (152). The copper content in tumor tissues of GC patients was positively correlated with patients' TNM stage and negatively correlated with patients' OS (overall survival) and DFS (disease-free survival) (153). ChenHuang et al. found that overloaded copper ions in GC could accelerate the lactylation process of non-histone protein METTL16-K229 by promoting the interaction of lactyltransferases AARS1/AARS2 with METTL16(a key regulator of copper death in gastric cancer), in order to ultimately lead to the development of Cuproptosis (116). Normal concentrations of copper are essential for human life processes. In contrast, high intracellular copper concentrations can cause cytotoxicity and even cell death. Therefore, copper absorption, distribution, and excretion in the body are tightly regulated by a copper-dependent protein network. Among them, Cu-binding proteins (CBP) are responsible for the transport of free copper into cells associated with cuproptosis. This is because GC cells produce intermediate metabolites and energy more through glycolysis than oxidative phosphorylation (113). Therefore, if the cuproptosis process is induced in GC cells, it might have a positive effect on tumor tissue invasion as well as metastasis. Currently relevant studies have identified CBP as an entry point for research with great clinical potential in the treatment and prognosis of GC patients (154). Moreover, TP53, one of the most commonly mutated genes in GC, is an important regulator affecting tumor metabolism, which can be involved in mediating cellular glycolysis and oxidative phosphorylation processes in order to increase the sensitivity of these two tightly coupled metabolic processes in cells to cuproptosis (155). Therefore, studies targeting TP53 and cuproptosis are of increasing interest to researchers. Currently, the research on disulfiram combined with copper in the treatment of GC has also made good progress. It has been shown that DSF/Cu complex can not only inhibit the tumor activity of GC cells by regulating the signaling pathways such as stress response, glycolysis, Wnt/β-catenin, etc., but also indulge the apoptosis of cells by participating in the reactive oxygen species (ROS)/mitogen-activated protein kinase pathway (156). And a regional study showed that the use of calcium channel blockers reduced the chances of developing GC to some extent, which may indicate a specific link between calcium signaling pathways and GC (157). The following are some of the research directions available. IL-15 is a cytokine that has an active role in the adaptive and intrinsic immune system of the body and in tumorigenesis. Some studies have found a correlation between copper death-related genes LIPT1, FDX1, MTF1 and IL-15 (with LIPT1 having the strongest correlation, R = 0.348). Numerous clinical trials have reported that drugs developed on the basis of IL-15, such as recombinant human single-chain IL-15 and IL-15 super agonists, have better efficacy in tumor therapy. Based on the relationship between IL-15 and cuproptosis-related genes, perhaps studies combining IL-15-related drugs with cuproptosis gene targets could provide new ideas and insights into the treatment of GC (158).

#### 5.3.1 H. pylori

**Figure 4:**
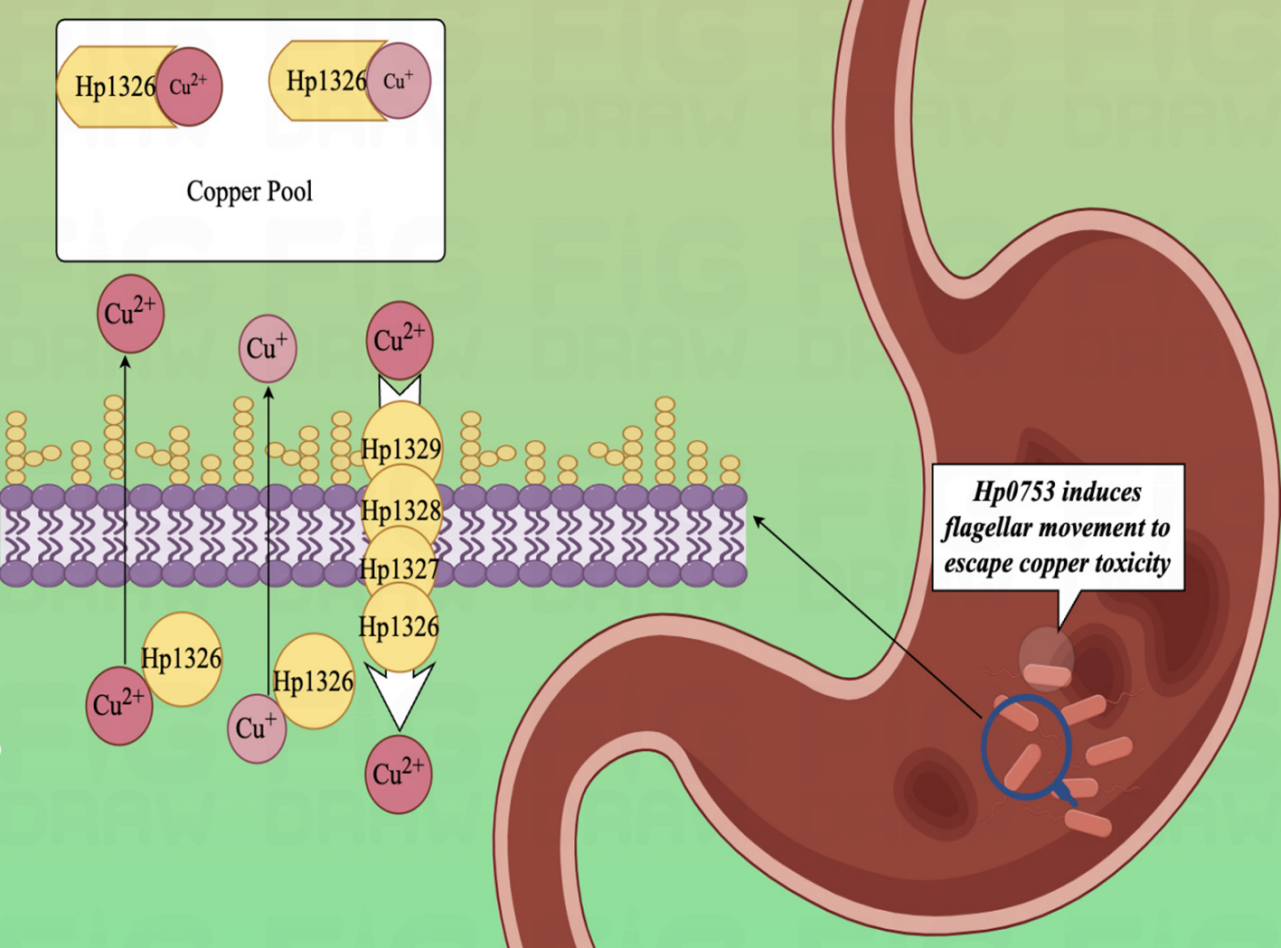
Diagram of the copper efflux mechanism.HpCrdA interacts with other H. pylori copper efflux pump proteins (CrdB, HP1328, and HP1329) to transport overloaded copper ions *in vivo* outside the H. pylori bacterium, which limits the cytoplasmic concentration of free copper ions and keeps them below toxic levels.

In the experimental study of copper binding by the H. pylori CrdA protein (HpCrdA), Ivana Kekez et al. found that CrdA selectively binds excess free copper ions in the form of Cu(I) and Cu(II), when the concentration of free copper ions in the cytoplasm of the H. pylori cell reaches a high value. Further, HpCrdA interacts with other H. pylori copper efflux pump proteins (CrdB, HP1328, and HP1329) to transport overloaded copper ions *in vivo* outside the H. pylori bacterium, which limits the cytoplasmic concentration of free copper ions and keeps them below toxic levels. And that process also has a positive response to external changes in the concentration of copper ions (159). Already in 2000, Waidner et al. presented this new system of copper externally discharged pumps (Czc system), which consists of the copper resistance determinants CrdA (HP1326), CrdB (HP1327), CzcB (HP1328) and CzcA (HP1329). With this system, H. pylori decrease copper-mediated production of toxic hydroxyl radicals (160, 161) and keep intracellular copper ion concentrations under toxic levels (**Figure 4**). Except, copper ions also induce the fliS gene in H. pylori, which together form the indispensable link to the production of an intact flagellum by H. pylori (162). Copper ions play an influential role in electron transport, oxidative enzymes, and hydroxylases, as an indispensable cofactor in the regulation of metal homeostasis in H. pylori (163, 164). As a clear class**I**causative organism of GC, H. pylori can lead to the development of gastric malignant tumors through the process of AG-GIM, so it has a pivotal role in the prevention and diagnosis of GC. Furthermore, *in vitro* experiments have shown that human GC cells (AGS) can cause up-regulation of the CAC and amino acid metabolism, by modulating the MTOR complex 1 (MTORC1) signaling pathway in the gastric epithelium and immune cells at 6 hours after infection (165, 166).

Considerable clinical and basic experimental studies have proven the efficacy of copper complexes or chelator drugs using copper as a carrier in the treatment of malignant tumors. Examples include combining disulfiram and copper ions for breast cancer (165), elesclomol-Cu^2+^ for the treatment of malignant tumors (111), etc. Based on the mechanism of interaction between copper and H. pylori, we may be able to find the Cuproptosis principle. Perhaps it can be further exploited to cause cytotoxicity and stress death of H. pylori, finding a new pathway to reduce the risk of GC development.

#### 5.3.2 Antiangiogenic direction

Angiogenesis is a physiological process that is co-regulated by pro-angiogenic factors and anti-angiogenic factors which is characterized by the sprouting of new blood vessels from pre-existing vessels. In mammals, the process of angiogenesis is generally dormant (148) and is only visible in pathological or rarely physiological states. In malignant aggressive tumors, including GC, however, it is one of the steps necessary for the growth and metastasis of tumor cells (167, 168).

The concept of Cu^2+^ pro-angiogenesis was first proposed by Professor McAuslan. In experiments on copper-induced phagocytosis of aortic endothelial cells, he found that mutant aortic endothelial cell lines were highly sensitive to copper. Under experimental conditions, endothelial cells are migrated by copper salts, traveling up to 24 μm in 1000 hours. The discovery of that phenomenon lays the foundation for the study of early neovascular event modeling systems (169).

VEGF-A is a crucial mediator in the regulation of vascular growth and development in pathological states. As a vascular endothelial cell mitogen, VEGF-A assumes a key role in regulating endothelial cell survival. VEGF-A promotes angiogenesis through interaction with vascular endothelial growth factor receptors 1 and 2 (VEGFR-1 and VEGFR-2) and co-receptors neuro phospholipids-1 and 2 (NRP-1 and NRP-2) (170).

Copper is an essential cofactor for the activation of metalloproteases (171, 172). It can be involved in regulating the affinity of angiopoietin for endothelial cells, in human physiological pH, by binding to angiopoietin molecules (eg. VEGF-A) (173). This drives the binding of angiopoietin to endothelial cells, causing endothelial cell (174) proliferation and migration. When pro-angiogenic molecules are more active than anti-angiogenic molecules, the angiogenic route is activated (175), and early angiogenesis occurs. Ultimately, it engages in tumor angiogenesis, accelerating cancer metastasis and deterioration (176).

**Figure 5:**
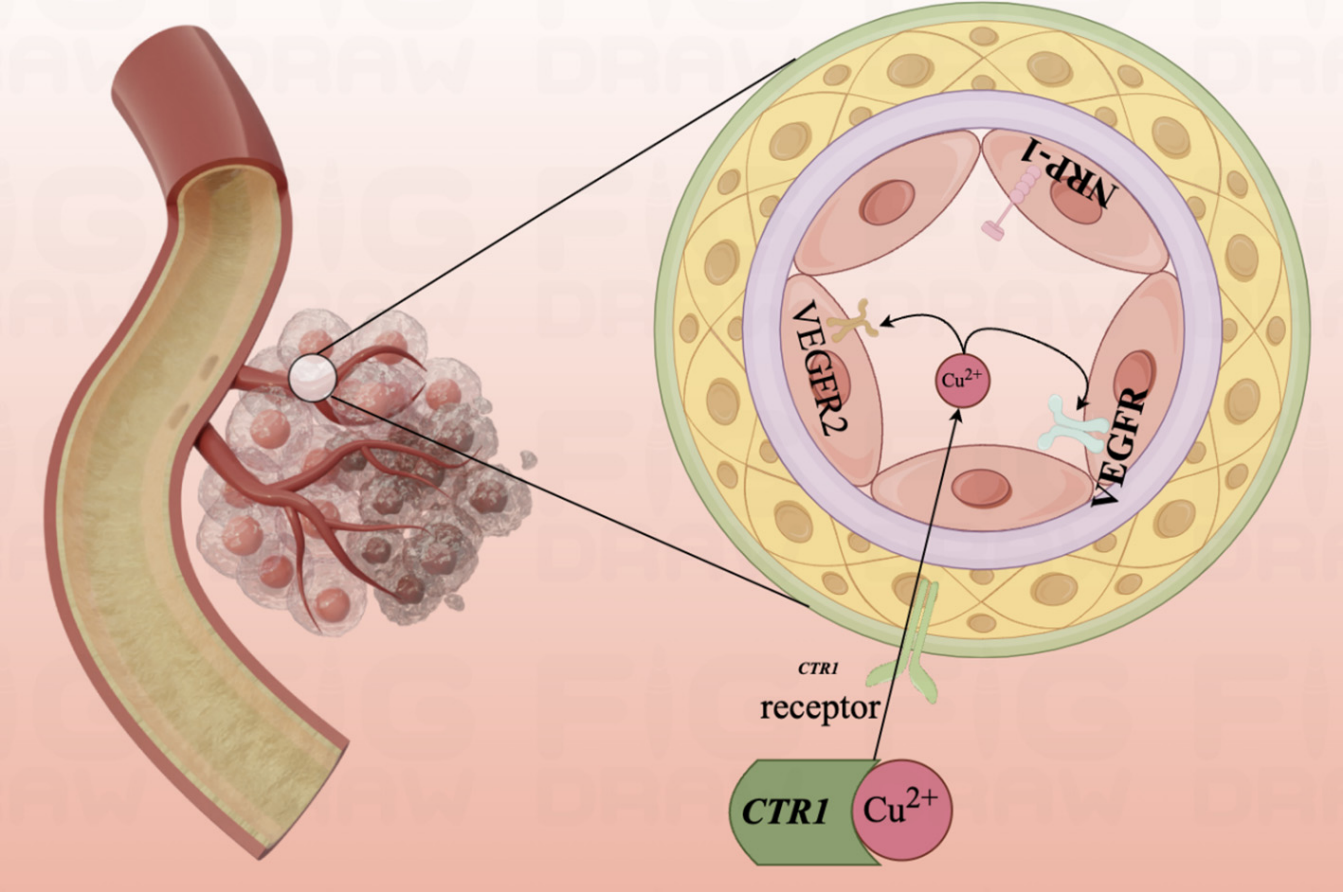
Diagram of the mechanism of copper ion involvement in angiogenesis.Mutant aortic endothelial cell lines are highly sensitive to copper, and copper ions promote the binding of angiopoietin to endothelial cells by binding to angiopoietin molecules, causing endothelial cell proliferation and migration. The angiogenic pathway is activated and early angiogenesis is turned on.

In 2023, Chunmei He et al. found that 11 out of 12 CRGs were up-regulated in endothelial cell expression, suggesting that CRGs may play a potential role in angiogenesis through lncRNA (177). Cuproptosis, as a landmark sign of cell death due to copper overload *in vivo*, is promising in inhibiting tumor angiogenesis and delaying tumor growth and metastasis and deserves the deep attention of researchers (**Figure 5**).

#### 5.3.3 TME immunization therapy

The term tumor microenvironment (TME) originated from the proposed ecological terminology (178), which is used to describe the internal and external environment of tumors or tumor cells with the ability of self-renewal and tumor driving (144).

TME consists of various cells (including immune cells, endothelial cells, inflammatory cells, fibroblasts, and lymphocytes), ECMs, vascular systems, and chemokines (179–181). The occurrence, evolution, and metastasis of tumors are intimately related to the structure and function of TME. Currently, in the field of cancer immunotherapy, the use of TME modulation strategies in the treatment of malignant tumors has attracted considerable attention (182). Immune cells in TME mediate innate and adaptive immune response processes. Innate immune cells have pro-tumor and anti-tumor properties. The adaptive immune system, activated by inherent immune cells, can specifically attack tumor cells and is considered the most effective method for eradicating tumors. Although innate immune cells can detect tumors and induce and amplify adaptive immune responses, this function is limited by the immunosuppressive microenvironment in TME (183). Whereas copper ions in the body can promote innate immunity by enhancing the presenting capacity of dendritic cells (DCs) and macrophages, along with the cytotoxicity of natural killer cells (NK cells) (184–186). Moreover, copper ions can stimulate the activation and proliferation of adaptive immune cells (187), reverse topical immune suppression (188), and play an essential role in the immunotherapy of tumors (189).

TME in hypoxia alters the functioning of the normal microenvironment, promotes tumor progression, and limits therapeutic efficacy (190). Besides affecting cancer cell metabolism directly, copper is also involved in cancer therapy by regulating hypoxia within the TME (191, 192). Copper reverses the poor responsiveness of conventional cancer immunotherapy by inducing a redox reaction with simultaneous oxygen production (177). Until TME is fully formed, the tumor is noticed by the immune system. Particularly, the majority of patients will not normally die from a tumor originating in the primary site, but from metastatic tumors and associated changes in vital organs.

During the unrestricted growth of tumor cells uncontrolled by the cell death program, the innate immune surveillance of the organism changes. As a few tumor cells are killed by innate immune cells, the remaining ones gain the ability to rapidly increase in value due to the expanded growth space. Tumor cells then enter a phase of homeostasis to avoid being destroyed by the body's immune cells (escape phase). At this stage, tumor cells escape from immune destruction through pathways such as inhibition of ligand (PD-L) expression or secretion of inhibitory molecules (TGF-β) (193). The highly immune inhibitory properties of TME, which is carefully woven from tumor tissue, play an extremely important role in the above process all through the process (194).

Regulating the expression levels of PD-L1 (195)and other genes in immune checkpoint genes (IGCs) (143), copper ions can participate in tumor immunosuppression and tumor immunotherapy during the process of tumor cell proliferation and immune escape.

Copper is a crucial micronutrient that mediates the phagocytic role of innate immune cells (196, 197), including neutrophils and macrophages, tumor cell proliferation, bacterial infections, and other processes, with the aim of maintaining a healthy state of the organism. However, in the types of diseases caused by copper deficiency in the body, we found that copper, as an indispensable metal, has a vital role in the development and differentiation of bone marrow cells. Copper deficiency *in vivo* induces neutropenia and anemia (198). And, neutrophils have been proven to have a pre-tumor cell phenotype within the TME owing to their tumor-associated macrophage-like role, and their tissue infiltration has been associated with poor tumor outcomes (199). It has been found that if copper deficiency occurs in the innate immune response before or during the elimination phase of tumor cells, the phagocytic step of immune cells will be impaired, leading to tumor cell progression (200). Moreover, studies suggest that the immune microenvironment may be closely related to cuproptosis in tumors (201).

Lin Jiang et al. Subtype classification and immunotype analysis of GC samples based on the consensus clustering method showed that most of the immune-related genes in cuproptosis were significantly up-regulated in the C2 subtype (202). Accordingly, the modification of TME by regulating the cancer immune environment has increasingly attracted the attention of researchers (203, 204).On the clinical outcome of bladder cancer and the score of the immune response experiment system, cuproptosis immune cell infiltration was found to be associated with bladder cancer prognosis, and high CD8^+^T cell infiltration predicted a positive prognosis (205). This implies that cuproptosis may be involved in the regulation of TME, especially CD8^+^T cells, thus contributing to tumor growth and progression. Regulatory T cells are involved in homeostatic regulation of the organism and tumor immune escape.GC cells recruit Regulatory T cells while inducing CD4 T naïve cells to differentiate into Regulatory T cells via TGF-β, which ultimately achieves immunosuppressive effects (206). Immune checkpoint inhibitors enhance the prognosis of GC of patients, among which PD-L1/PD-1 arresters have excellent anti-tumor immunological effectiveness (207) .This also brings a new dawn for exploring the mechanism of cuproptosis in the immunotherapy of gastric malignant tumor microenvironment. Recent studies have also shown that cuproptosis is an important factor in tumor resistance to immunotherapy and antitumor therapy. Therefore, exploring the intrinsic mechanisms of immunomodulatory factors and cuproptosis in tumorigenesis and development, and further delving into the specific mechanisms cuproptosis in the overall prognosis, biomarkers (diagnostics), and immunotherapy of GC patients may provide new ideas for immunotherapy in the microenvironment of gastric malignant tumors.

#### 5.3.4 Tumor metastasis

Cancer is a disease in which a group of tumor cells constantly proliferate, leading to the formation of tumors. It has been shown that the copper-exporting protein ATP7A promotes tumor cell growth by increasing copper levels in the cells (208). These tumor cells can invade the surrounding tissues and spread to distant organs (209, 210) through a cascade of metastasis (211, 212). According to reports, over 90% of cancer-related deaths are caused by the metastatic spread of cancer cells (213).

Copper ions affect the behavior of tumor cells in various ways during their invasion and metastasis. It can profoundly engage in the invasion and migration of tumor cells by mediating the connection between ECMs and cytoskeleton (214), as well as acting as gradient forming agent and intracellular messenger (215). As the central molecule in tumor cell migration, integrins can be activated by a combination of divalent metal ions relevant to the cell surface, such as iron, zinc, copper, or ECMs proteoglycans. They may lead to tumor cell migration. Additionally, as a coenzyme, a factor necessary for the cross-linking of collagen and elastin fibers (216), a copper ion can modify the extracellular matrix and create a suitable environment for tumor cell metastasis by enhancing the activities of lysyl oxidase (LOX) and lysine oxidase-like enzyme (LOXL) (116, 217). Recent studies have found that copper concentration in mucinous adenocarcinoma tissues in GC has a positive correlation with invasive fingers such as lymph node metastasis (218, 219). In addition to LOX, copper activates ERBB2-driven cellular motility protein mediator 1 (Memo1), which activates the phosphatidylinositol-3 kinase/protein kinase B (PI3K/Akt) signaling pathway by interacting with the insulin receptor substrate 1 to promote EMT, clearing Cu into the secretory pathway and reducing ROS formation. And, in experiments related to the migration and metastasis of breast cancer cells, we found that the cell movement protein MEMO, which is catalyzed by Cu^2+^ oxidase activity, has a clear ability to promote cell migration of breast cancer. Above all, Cu^2+^ may have the potential to influence cancer cell metastasis (220).

Nikos K Karamanos et al. used KEGG pathway enrichment and GO annotation to show that significantly differentiated genes (DEGs) in cuproptosis are mainly involved in the construction of extracellular matrix organization, cell-cell junctions, and collagen-containing extracellular matrix components (221). How copper overload *in vivo* induces a protein-toxic stress response and leads to programmed cell death, may give us new insights and inspiration in the study of Cu^2+^ in the treatment of malignant tumor cell metastasis.

#### 5.3.5 Chemotherapeutic resistance

In GC we find that copper levels are significantly elevated, and this is more common in more malignant and advanced stages of GC (116). Related Researchers see the potential link between copper ions and chemotherapy resistance in tumor patients, due to higher serum copper levels in chemotherapy-resistant patients compared to those responding to chemotherapy (222). Jing Jin et al. also found that in tumor tissues, physiologically high copper can lead to chemotherapy resistance in patients by promoting DNA double-strand break repair in the role of copper in drug-resistant murine and human tumors tumor cells (223). Many genetic and biochemical studies have also shown that the copper transporter proteins ATP7A and ATP7B can promote platinum drug resistance by controlling their uptake and export from tumor cells (224).

Currently, platinum-based drugs are often used clinically in the treatment of cancer patients. The main challenge affecting its therapeutic efficacy is chemotherapy resistance, which is one of the main reasons for the failure of anti-cancer treatments (225). Unlike exogenous platinum drugs that often lead to the up-regulation of glutathione (GSH) stress in tumor cells and thus lead to chemotherapy resistance in tumor patients, a team of researchers has found that Diethyldithiocarbamic Acid Copper Salt (CuET) reverses cisplatin resistance in non-small-cell lung cancer through a cuproptosis mechanism (226). They also bring new inspiration for inducing cuproptosis in GC to achieve the reversal of platinum drug resistance (227). Since glutathione and glucose depletion enhance the sensitivity of cancer cells to cuproptosis mediated by GOx@ [Cu(tz)] (228), targeted therapy against cuproptosis might be a new therapeutic strategy to overcome chemotherapy resistance.

### 5.4 Cuproptosis and prognosis of GC

Currently, immunology-based studies on the tumor microenvironment aimed at exploring the relationships between CRGs and the prognosis of GC have made promising progress.

In addition, studies have shown that elevated copper levels are seen in a variety of human cancers, such as colorectal, breast, liver and lung cancers. Therefore, more and more studies have begun to focus on the relationship between cuproptosis and these cancers, and many studies have shown that CRGs are closely associated with the prognosis of various types of cancers (156). Xichun Hu et al. established a nomogram based on the combination of TME-based CRGs risk score and clinicopathologic features to predict the risk associated with the prognosis of patients with GC, and found that CRGs was significantly correlated with TME as well as the prognostic assessment of GC (156). This was also confirmed by the study of Wang Ning et al. The study further found that the level of immune infiltration of tumor tissues was negatively correlated with the expression level of CRGs as well as the prognosis of patients (207). Jin Liu et al. also found that theCRGs SERPINE1 is associated with immune cell infiltration. This gene can be used to maintain proliferative signaling in tumors and inhibit apoptosis through an inhibitory immune microenvironment. Abnormal expression of this gene in GC tissues is closely associated with poor prognosis in GC patients. The team further found that some statins may affect the expression of SERPINE1 through the TGF-β pathway. c-Met, a receptor tyrosine kinase that promotes tumor cell proliferation, invasion, and migration, is aberrantly expressed in a variety of tumor cells. And SGX523, as a c-Met inhibitor, may provide new ideas for the treatment of GC patients with aberrant expression of SERPINE1. SERPINE1 as a prognostic biomarker and a potential therapeutic target in GC also deserves further study by researchers (229). Jie Liet al. showed that cuproptosis-related immune genes (ANOS1, CTLA4, ITGAV, CXCR4, NRP1, FABP3, and LGR6) were up-regulated in GC tissues, and GC patients had poor prognosis. Among them, NRP1, CXCR4, LGR6, and CTLA4 were abnormally expressed in GC tissues and associated with cuproptosis-related gene FDX1. It is suggested that CRGs may be involved in the development and prognosis of GC through immune regulation. The results of this study reflect the increasingly important role of immune checkpoint inhibitor therapy in the comprehensive treatment of GC (230). The prognosis of GC patients is related to the infiltration of immune cells within the tumor and the response to immunotherapy, in which DNA methylation of tumor cells is also one of the factors affecting the prognosis of GC patients. Studies have shown that CRGs FDX1, LIAS and MTF1 are associated with multiple types of immune cell infiltration, which can be used as potential prognostic biomarkers for GC patients. This study provides new strategies for immune-targeted therapy in GC patients (118).

In addition, lncRNAs(examples include LINC01150,SNAP25-AS1,LINC00571 and HAGLR) (231) associated with cuproptosis could also accurately predict the prognosis of GC patients. It may have a better predictive effect than a single biomarker. However, the specific mechanism needs to be further explored (232).

## 6 Conclusions

As an indispensable coenzyme factor for various life activities in the human body, the intake of copper by the human body mainly comes from food. After digestion of copper-containing food by the stomach, absorbed by small intestinal copper transport protein 1 (CTR1) or solute carrier family 31 member 1 (SLC31A1) (233), exists in the body in the form of Cu^+^ and Cu^2+^ (234). The absorbed Cu^2+^ is reduced to Cu^+^ by reductase upon binding to divalent metal transporter 1 (DMT1) and then binds to transmembrane copper transporter 1 (CTR1) to enter the cells (235). It goes through different modes of transport to bind to certain copper proteins (236). Eventually, it functions in various tissues and organs of the human body. However, as the intracellular copper concentration reaches a certain threshold, it also triggers the FDX1 gene to reduce Cu^2+^ to the more toxic Cu^+^ (88), leading to cuproptosis. To prevent the accumulation of harmful free copper in human cells (116), copper can maintain its concentration in cells within the physiological level through an active homeostasis mechanism (237).

Cancer, has increasingly become a global discussion hotspot, with a high level of research and exploration on GC. Copper ions have received substantial evidence indicating that their concentration disorders can occur in various cancers, including GC. Considering that the sites of copper absorption are mainly in the stomach and small intestine, gastrointestinal tumors are particularly suitable for studying the mechanisms associated with cuproptosis and GC (238).Copper ions are widely involved in the occurrence and development of GC. According to the cuproptosis view, excess intracellular copper can reduce Cu^2+^ to Cu^+^ with great-er toxicity under the joint action of mitochondrial Fe-S cluster protein and ferredoxin 1 (FDX1). Furthermore, copper can bind directly to DLAT and contribute to the disulfide-dependent aggregation of lipoylated DLAT, eventually leading to cuproptosis. With high FDX1 protein levels and copper in GC, cuproptosis may be more likely to be triggered. It offers a potential therapeutic strategy for GC, especially for a malignant tumor - mucinous adenocarcinoma. Yuan Chen et al. (116)conducted a meta-analysis found that the genes LIAS, DLAT, DBT, and PDHA1 exhibit extensive differential expression in Crohn's disease (CD), ulcerative colitis (UC), celiac disease (CEL), and IBD-induced cancer (IBD-CA). According to molecular docking results, methotrexate shows the highest binding affinity to the main chain of copper apoptosis-related regulatory factors. Weichen Wang et al. (238) collected glioma datasets from TCGA, GEO, and CGGA databases, analyzed CRGs (CRG) using the Robust Multichip Average (RMA) algorithm, and calculated CuproptosisScore using multivariate Cox regression analysis. They found that patients with higher CuproptosisScore also have higher WHO grades and poorer prognosis, while patients with lower CuproptosisScore are more likely to have IDH mutations or MGMT methylation. In the high CuproptosisScore group, PIK3CA, MUC16, NF1, TTN, TP53, PTEN, and EGFR exhibit high mutation frequencies, and immune infiltration levels increase with increasing CuproptosisScore. Patients with high CuproptosisScore may respond better to anti-PD-1 therapy. Lianhui Sun et al. (116) published a study revealing significantly elevated copper levels in GC. Through meta-analysis and enrichment gene screening, they identified METTL16 as a key mediator of copper apoptosis in GC through m6A modification on FDX1-mRNA. High copper levels promote non-histone protein METTL16-K229 acylation by increasing the interaction between potential aminoacyl-tRNA synthetases AARS1/AARS2 and METTL16, ultimately leading to copper apoptosis. Copper and lactate concentrations in gastric tumors (especially malignant tumors) are higher than in normal tissues. Combining the copper ion carrier disulfiram and SIRT2-specific inhibitor to induce copper apoptosis significantly enhances the efficacy of GC treatment. These research findings provide a comprehensive understanding of the mechanisms underlying copper apoptosis initiation and execution and propose a promising therapeutic strategy for GC.

The current research on the association between Cuproptosis and GC is still confined to applying CRGs for constructing prognostic risk models for GC. There is still a lack of research on the mechanism of cuproptosis in the occurrence and development of GC, without its application in the treatment of GC. Therefore, this article reviews the enrichment and expression of CRGs in the occurrence and development of GC, the mechanism of cuproptosis and GC occurrence and development, and the prospect of treatment methods for GC using the proptosis perspective. The objective is to provide a feasible basis for utilizing the concept of cuproptosis in GC research, as well as to provide some new perspectives for future research on GC prevention and treatment.

Many studies in recent years have shown that cuproptosis is closely associated with ROS and inflammation and triggers other forms of cell death, including apoptosis, pyroptosis, and ferroptosis. It has been found that intracellular mitochondrial ROS levels are elevated and induce apoptosis, whereas ROS inhibitors rescue cell viability. Further studies revealed that ros-induced apoptosis is dependent on the sustained activation of the pro-apoptotic mitogen-activated protein kinase (MAPK) pathway (cJun n-terminal kinases [JNKs] and p38), which regulates the phosphorylation of pro- and anti-apoptotic proteins in mitochondria. Similarly, disulfiram-cu^2+^ complexes can induce ROS production, which in turn activates the downstream apoptosis-related JNK and p38MAPK pathways to induce apoptosis in breast cancer cells. From an inflammatory perspective, activation of nucleotide-binding oligomerization structural domains, leucine-rich repeat sequences, and pyrin structural domain-containing protein 3 (NLRP3) inflammasome pathways were found to introduce copper-mediated macrophage pyroptosis. That means it's an inflammatory form of lysogenic programmed cell death. Similar results were found in copper oxide nanoparticles (CuONPs)-treated mouse macrophages showing elevated levels of pro-inflammatory factors including NLRP3, caspase-1, and interleukin (IL)-1β. In the acidic environment of the lysosome, CuONPs attack the lysosome by releasing copper ions, leading to the release of histone B, which directly mediates the activation of the NLRP3 inflammasome. In addition, CuONP exposure triggered macrophages to express pro-il -1β through activation of myeloid differentiation factor 88 (MyD88)-dependent toll-like receptor 4 (TLR4)/nuclear transcription factor κB (NF-κB) cascade response. This is another typical pathway of NLRP3 inflammasome activation.

However, there is still a lack of systematic studies on other roles of copper in mitochondria, such as its induced cell death phenotypes and specific clinical treatments for related chelators. At the same time, the crosstalk between its mediated programmed cell death and other metal-induced cytotoxicity could not be further investigated in detail. This has prompted studies of the mechanisms involved in the onset of relevant cuproptosis in GC development, which must be tailored to the toxicology of metal toxicity in cells within the various layers of tumor tissue at different stages of differentiation or progression.

Above all, considering the correlation between cuproptosis and GC, we can predict that new treatment methods may become the wave of GC treatment. When that happens, GC patients will enjoy more effective and personalized treatment strategies, better prospects, and quality of life, and the new diagnostic and treatment approach will bring a new dawn to lighten the financial burden of those patients.
